# A historical review of surgery for peritonitis secondary to acute colonic diverticulitis: from Lockhart-Mummery to evidence-based medicine

**DOI:** 10.1186/s13017-017-0120-y

**Published:** 2017-03-09

**Authors:** Roberto Cirocchi, Sorena Afshar, Salomone Di Saverio, Georgi Popivanov, Angelo De Sol, Francesca Gubbiotti, Gregorio Tugnoli, Massimo Sartelli, Fausto Catena, David Cavaliere, Renata Taboła, Abe Fingerhut, Gian Andrea Binda

**Affiliations:** 1 Department of General Surgery, University of Perugia, Terni Hospital, Terni, Italy; 2 Department of General Surgery, Cumberland Infirmary, Carlisle, UK; 3 General (Colorectal) Emergency and Trauma Surgery Service, Maggiore Hospital Regional Emergency Surgery and Trauma Center – Bologna Local Health District, Bologna, Italy; 4 Military Medical Academy, Sofia, Bulgaria; 5 Department of Surgery, Terni Hospital, Terni, Italy; 6 Department of Surgery, University of Ferrara, Ferrara, Italy; 7 Department of Surgery, Macerata Hospital, Macerata, Italy; 8 Department of Emergency Surgery, Parma Hospital, Parma, Italy; 9 Department of Surgery, Forlì Hospital, Forlì, Italy; 10 Department of Gastrointestinal and General Surgery, Medical University of Wrocław, Wrocław, Poland; 11 Section for Surgical Research, Department of Surgery, Medical University of Graz, Graz, Austria; 12 First Department of Surgery, Hippokration University Hospital, University of Athens, Athens, Greece; 13 Department of Surgery, E.O. OspedaliGalliera, Genova, Italy

**Keywords:** Acute diverticulitis, Emergency surgery, Perforated diverticulitis, Laparoscopic lavage, Colorectal surgery, Acute care surgery, Hartmann resection

## Abstract

The management of patients with colonic diverticular perforation is still evolving. Initial lavage with or without simple suture and drainage was suggested in the late 19th century, replaced progressively by the three-stage Mayo Clinic or the two-stage Mickulicz procedures. Fears of inadequate source control prompted the implementation of the resection of the affected segment of colon with formation of a colostomy (Hartman procedure) in the 1970’s. Ensuing development of the treatment strategies was driven by the recognition of the high morbidity and mortality and low reversal rates associated with the Hartman procedure. This led to the wider use of resection and primary anastomosis during the 1990’s.

The technique of lavage and drainage regained popularity during the 1990’s. This procedure can also be performed laparoscopically with the advantage of faster recovery and shorter hospital stay. This strategy allows resectional surgery to be postponed or avoided altogether in many patients; and higher rates of primary resection and anastomosis can be achieved avoiding the need for a stoma. The three recent randomized controlled trials comparing laparoscopic peritoneal lavage alone to resectional surgery reported inconsistent outcomes.

The aim of this review is to review the historical evolution and future reflections of surgical treatment modalities for diffuse purulent and feculent peritonitis. In this review we classified the various surgical strategies according to Krukowski et al. and Vermeulen et al. and reviewed the literature related to surgical treatment separately for each period.

## Background

Colonic diverticulosis is an increasingly common clinical condition in Western Europe and North America [1]. Most people with colonic diverticula will remain completely asymptomatic. However, 10–20% of patients with diverticulosis will manifest symptoms and signs of illness. Symptomatic diverticular disease (DD) can be separated into DD without inflammation (75%) and with inflammation or diverticulitis [2]. The former can also be painful in spite of the lack of inflammation [2]. Acute diverticulitis is defined as acute inflammation of a colonic diverticulum [3]. Peridiverticular and pericolic infections are a result of microscopic or macroscopic perforation of a diverticulum. The spectrum of acute diverticulitis varies between mild diverticulitis and diffuse feculent peritonitis [4]. Starting in 1978, Hinchey’s classification has been used for the staging of complicated diverticulitis [5]. Several modifications of Hinchey’s traditional classification have been proposed [6, 7] (Table 1).

**Table 1:** Hinchey classification of perforated diverticulitis

Hinchey stage	Features of disease
Stage I^a^	Diverticulitis with a pericolic abscess
Stage II^b^	Diverticulitis with a distant abscess (this may be retroperitoneal or pelvic)
Stage III	Purulent peritonitis
Stage IV	Fecal peritonitis

^a^Stage I has been divided into Ia (phlegmon) and Ib (confined pericolic abscess)

^b^Stage II has been divided into distant abscesses amendable for percutaneous drainage (stage IIa) and complex abscesses associated with a possible fistula (stage IIb)

The European Association for Endoscopic Surgeons (EAES) classification system divides the severity of diverticulitis into three different grades of disease [8] (Table 2). In-hospital mortality after emergency surgery for acute perforated diverticulitis is high (29%) and the Hinchey stage has been found to be a significant predictive factor for mortality [9].

**Table 2:** European Association for Endoscopic Surgeons classification system for colonic diverticulitis (1999)

Grade of disease	Description	Clinical state of the patient
I	Symptomatic uncomplicated disease	Pyrexia, abdominal pain, CT findings consistent with diverticulitis
II	Recurrent symptomatic disease	Recurrence of Grade I
III	Complicated disease	Bleeding, abscess formation, phlegmon, colonic perforation, purulent and fecal peritonitis, stricture, fistula and obstruction

The main cause for the high mortality rate is due to sepsis and prognosis is associated with severity of peritonitis as measured by scoring systems such as the Acute Physiology and Chronic Health Evaluation (APACHE), Mannheim peritonitis index (MPI) and Sequential Organ Failure Assessment (SOFA) [10, 11]. SOFA score was developed to assess organ dysfunction and morbidity and in contrast to APACHE II it allows serial follow-up [11]. The predictive value for death at admission and after 72 h is 75% and 84% respectively [12].

According to current practice guidelines, patients with generalized peritonitis should undergo emergency surgery, as suggested by Mikulicz in 1889 [13]. However, despite intensive research carried out during the last century, the best treatment algorithm is yet to be determined.

The aim of this review is to expose the historical evolution and future reflections of surgical treatment modalities for purulent and feculent peritonitis.

## Methods

Surgical strategies were stratified according to the classifications proposed by Krukowski et al. and Vermeulen et al. [14, 15] (Table 3). We reported the essential literature relating to surgical treatment separately for each decade from 1900 to 2016. Only the highest grade of evidence published for each topic was noted for each period. The hierarchy of evidence grading system proposed by the Centre for Evidence-Based Medicine of Oxford was used [16].

**Table 3:** Operative Procedures

Conservative: perforated colon retained in peritoneal cavity	
1. Suture of perforation	
2. Drainage	
3. Transverse colostomy	
4. Caecostomy	
5. Any combination of 1–4	
Radical: perforated colon eliminated from peritoneal cavity	
1. No resection	
• Exteriorization	
2. Resection	
a. Without anastomosis	
• Hartmann’s procedure	
• Sigmoid resection with mucous fistula	
• Paul-Mickulicz procedure	
b. With anastomosis	
• Without defunctioning stoma	
• With defunctioning stoma	

**Fig. 1:**
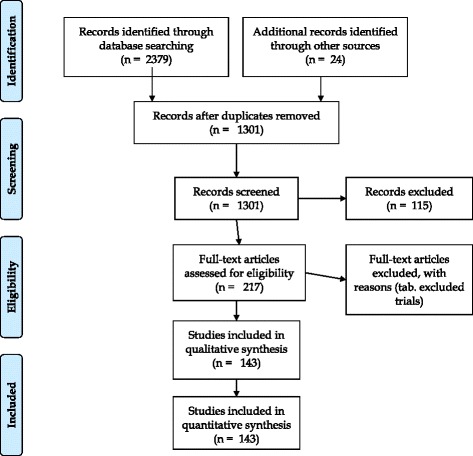
Prisma flow diagram of the study

This systematic review was performed in accordance with the Preferred Reporting Items for Systematic Reviews and Meta-analyses (PRISMA) standards (Fig. 1) [17]. We conducted a systematic literature search using PubMed employing the terms perforated OR peritonitis AND diverticulitis; we search in the published papers from January 1st 1990 to May 2016 [18]. The PubMed function “related articles” was used to broaden each search, and the reference list of all potentially eligible studies was analysed. In addition, a manual search method including the Science Citation Index Expanded, Scopus and Google Scholar databases was performed. After this initial screening process, two authors (RT, RC) independently assessed eligibility of full-text papers. The final decision on eligibility was reached by consensus between the two authors. When multiple articles were published from a single study group and where overlapping study periods were reported, only the most recent article was considered to avoid duplication of data. Data were extracted based on an intention-to-treat principle. Any disagreement was resolved through discussion with a reassessment of the data and/or by involving a senior author.

## Results

The PRISMA flow diagram for systematic reviews is presented in Fig. 1. We identified 2,403 publications using the literature search strategy described above and additional searches through other sources. After excluding 2,186 records following the duplicate removed and the review of the titles and abstracts, 217 abstracts eligible for full-text evaluation remained. After full-text assessment we identified 143 publications that fulfilled the inclusion criteria.

Surgical treatment of acute generalized peritonitis from diverticulitis was described as early as 1910 by Lockhart-Mummery [19] who advocated washing the peritoneum and abdominal drainage, combined, if possible, with suture of the colonic perforation (Tables 4 and 5) [19–113].

**Table 4:** Included studies published up to 1980

Author Year	Center Nation	Years of study	Level of evidence	Patients	Treatment
Lockart-Mummery 1910	St Mark Hospital (London) England	1910	5	Peritonitis from perforated diverticulitis	Toilette of the peritoneum and abdominal drainage ± suture
Judd and Pollock 1924	Mayo Clinic (Rochester, Minn.) USA	1907–24	4	Perforated diverticulitis	Toilette of the peritoneum and abdominal drainage ± suture
Wheeler 1930	Dublin Ireland	1930	5	Peritonitis from perforated diverticulitis	Caecostomy associated at Mikulicz’ procedure
Rankin 1930	Mayo Clinic (Rochester, Minn.) USA	1916–28	4	Perforated diverticulitis	Resection of diverticulun and suture Three-stage procedure: proximal colostomy. resection of the sigmoid colon, closure of the colostomy after a few weeks
Eggers 1931	Lenox Hill Hospital (New York) USA	1931	4	Peritonitis from perforated diverticulitis	Toilette of the peritoneum and abdominal drainage
Conway 1931	New York Hospital (New York) USA	NR	4	Peritonitis from perforated diverticulitis	Mikulicz’ procedure
Lockart-Mummery 1934	St Mark Hospital (London) England	1934	5	Peritonitis from perforated diverticulitis	Toilette of the peritoneum and abdominal drainage ± suture
Hunt 1934	Los Angeles USA	NR	5	Perforated diverticulitis	Drainage and colostomy - Mikulicz’ procedure
Eggers 1941	Lenox Hill Hospital (New York) USA	1938–39	4	Peritonitis from perforated diverticulitis	Toilette of the peritoneum and abdominal drainage – Drainage and colostomy –Mikulicz’ procedure
Smithwick 1942	Massachusetts General Hospital (Boston) USA	1925–42	4	Peritonitis from perforated diverticulitis	Toilette of the peritoneum and abdominal drainage ± suture - Drainage and colostomy - Mikulicz’ procedure
Pemberton 1947	Mayo Clinic (Rochester, Minn.) USA	1908–45	4	Perforated diverticulitis	Drainage and colostomy - Mikulicz’ procedure
Arnheim 1950	Mount Sinai Hospital (New York) USA	1927–37	4	Peritonitis from perforated diverticulitis	Drainage and colostomy - Mikulicz’ procedure
Boyden 1950	Portland, USA	NR	4	Peritonitis from perforated diverticulitis	Drainage and colostomy - Mikulicz’ procedure - Hartman procedure
Hughes 1952	Monash University (Melburne) Australia	1941–51	4	Peritonitis from perforated diverticulitis	Drainage – Drainage and colostomy – Exteriorization of the affected loop
Lloyd-Davies 1953	Kent and Canterbury Hospital (Canterbury) England	NR	5	Peritonitis from perforated diverticulitis	Drainage and colostomy
Welch 1953	Massachusetts General Hospital (Boston) USA	1942–53	4	Perforated diverticulitis	Drainage and colostomy
Lewis 1953	Yale Medical School (New Haven) USA	NR	4	Perforated diverticulitis	Drainage and colostomy
Edwards 1954	Surgeon to King’s College Hospital, (London) England	NR	5	Peritonitis from perforated diverticulitis	Drainage – Drainage and colostomy – Exteriorization of the affected loop
Scarborough 1954	Stanford University (San Francisco) USA	1954	5	Peritonitis from diverticulitis	Drainage and colostomy
Welch 1955	Massachusetts General Hospital (Boston) USA	1942–55	4	Perforated diverticulitis	Drainage and colostomy
Gregg 1955	State University of New York (New York) USA	NR	4	Perforated diverticulitis	Resection with anastomosis and covering stoma
Ransom 1956	University of Michigan (Ann Harbor) USA	1934–51	4	Peritonitis from perforated diverticulitis	Drainage and colostomy
Bacon 1956	University of Pennsylvania (Philadelphia) USA	1940–1955	4	Perforated diverticulitis	Drainage – Drainage and colostomy –Mikulicz’ procedure - Exteriorization of the affected loop
Belding 1957	Medical Clinic (Riverside) USA	1951–52	4	Peritonitis from perforated diverticulitis	Resection with anastomosis
MacLaren 1957	Royal Infirmary, (Edinburgh) United Kingdom	NR	4	Perforated diverticulitis	Resection with anastomosis
Ryan 1958	St Vincent’s (Melbourne) Australia	1954–56	4	Peritonitis from perforated diverticulitis	Drainage - Drainage and colostomy - Resection with anastomosis
McGregor 1958	Temple University, (Philadelphia) USA	1940–57	4	Peritonitis from perforated diverticulitis	Drainage and colostomy
O’Brein 1959	Hamilton General Hospital (Ontario) Canada	1952–59	4	Perforated diverticulitis	Drainage and colostomy
Brown 1960	Western Infirmary, (Glasgow) England	1945–56	4	Peritonitis from perforated diverticulitis	Drainage – Drainage and colostomy –Mikulicz’ procedure
Greig 1960	South Down Group of Hospitals Northern Ireland	1952–59	4	Peritonitis from perforated diverticulitis	Drainage – Drainage and colostomy
Boyden 1961	The Portland Clinic, (Portland) USA	NR	5	Peritonitis from diverticulitis	Hartmann’ procedure
Donald 1961	(Birmingham) USA	NR	4	Peritonitis from diverticulitis	Drainage and colostomy
Beard 1961	St. George Hospital (London) England	NR	4	Peritonitis from diverticulitis	Drainage and colostomy
Stauton 1962	Morriston Hospital (Swansea) England	1955–59	4	Peritonitis from diverticulitis	Exteriorization of the affected loop
Estrada 1962	Vancouver General Hospital (Vancouver) Canada	NR	4	Peritonitis from diverticulitis	Drainage – Drainage and colostomy
Hughes 1963	Monash University (Melburne) Australia	1951–61	4	Peritonitis from diverticulitis	Drainage – Drainage and colostomy – Exteriorization of the affected loop
Linder 1962	Brooklyn Women’s Hospital (New York) USA	NR	5	Perforated diverticulitis	Exteriorization of the affected loop
Hartley 1964	Addenbrooke’s Hospital Cambridge (London) England	1950–60	4	Perforated diverticulitis	Drainage – Drainage and colostomy
Large 1964	Royal Berkshire Hospital (Reading) England	NR	4	Perforated diverticulitis	Resection with anastomosis
Madden 1965	St Clare Hospital (New York) USA	1949–65	4	Perforated diverticulitis	Drainage and colostomy - Resection with anastomosis and covering stoma
Cochrane 1965	Fulham Hospital (London) England	1965	5	Perforated diverticulitis	Drainage and colostomy - Exteriorization of the affected loop
Dawson 1965	King’s College Hospital (London) England	1953–58	4	Peritonitis from diverticulitis	Drainage and colostomy –Mikulicz’ procedure - Exteriorization of the affected loop
Bacon 1966	University of Pennsylvania (Philadelphia) USA	NR	4	Peritonitis from diverticulitis	Drainage and colostomy
Smiley 1966	University of South California (Los Angeles) USA	1961–65	4	Peritonitis from diverticulitis	Drainage and colostomy - Mikulicz’ procedure – Hartmann’ procedure
Bolt 1966	West Middlesex Hospital, (London) England	1948–57	4	Peritonitis from diverticulitis	Drainage and colostomy - Exteriorization of the affected loop
Byrne 1966	St. Vincent Hospital, (Los Angeles) USA	1962–66	4	Perforated diverticulitis	Hartmann’ procedure
Watkins 1966	Washington University USA	1960–66	4	Peritonitis from diverticulitis	Exteriorization of the affected loop
Moseley 1966	Peter Bent Brigham Hospital (Boston), USA	1945–63	4	Peritonitis from diverticulitis	Drainage and colostomy - Hartmann’ procedure
Giffin 1967	Barnes Hospital (St. Louis) USA	1956–66	4	Peritonitis from diverticulitis	Drainage and colostomy - Exteriorization of the affected loop - Hartmann’ procedure - Resection with anastomosis
Levy 1967	University of Pennsylvania (Philadelphia) USA	1953–64	4	Peritonitis from diverticulitis	Drainage and colostomy - Exteriorization of the affected loop - Hartmann’ procedure
Localio 1967	New York Medical Center (New York) USA	NR	4	Peritonitis from diverticulitis	Drainage and colostomy - Mikulicz’ procedure
Colcock 1968	Lahey Clinic (Boston) USA	1947–67	4	Perforated diverticulitis	Drainage and colostomy - Mikulicz’ procedure – Hartmann’ procedure
Roxburgh 1968	Middlesex Hospital, (London) England	1964–67	4	Peritonitis from diverticulitis	Mikulicz’ procedure – Hartmann’ procedure - Resection with anastomosis and covering stoma
Rodkey 1969	Massachusetts General Hospital (Boston) USA	NR	4	Perforated diverticulitis	Drainage and colostomy - Mikulicz’ procedure
Moore 1969	Exter Hospital (Exeter) England	1960–67	4	Perforated diverticulitis	Hartmann’ procedure
Dandekar 1969	New Rochelel Hospital (New York) England	1960–66	4	Peritonitis from diverticulitis	Drainage and colostomy - Exteriorization of the affected loop - Hartmann’ procedure - Resection with anastomosis
Fenger 1969	Kommunehospitalet (Copenhagen) Denmark	1950–67	4	Perforated diverticulitis	Drainage and colostomy - Hartmann’ procedure
Reiss 1969	Meir Hospital (Kfar Saba) Israel	NR	4	Perforated diverticulitis	Drainage, colostomy and suture
Tagart 1969	Newmarket Community Hospital (Newmarket) England	1962–65	4	Peritonitis from diverticulitis	Drainage - Drainage and colostomy - Exteriorization of the affected loop - Hartmann’ procedure - Resection with anastomosis - Resection with anastomosis and covering stoma
Botsford 1969	Harvad Medical School (Boston) USA	1950–67	4	Peritonitis from diverticulitis	Drainage and colostomy - Hartmann’ procedure - Mikulicz’ procedure
Mitty 1969	St Vincent Hospital (New York) USA	1958–67	4	Perforated diverticulitis	Drainage and colostomy
Killingkack 1970	Audit Australia	1967–69	4	Peritonitis from diverticulitis	Drainage - Drainage and colostomy - Exteriorization of the affected loop - Hartmann’ procedure - Mikulicz’ procedure
Garnjobst 1970	Department of Surgery Providence Hospital (Portland) Oregon	1954–1969		diverticulitis	
Reilly 1970	Plymouth General Hospital, (Plymouth) England	NR	5	Peritonitis from diverticulitis	Drainage – Drainage and colostomy- Hartmann’ procedure
Ponka 1970	Henry Ford Hospital Detroit USA	1963–67	4	Peritonitis from diverticulitis	Drainage and colostomy- Hartmann’ procedure
Barabas 1971	Royal Postgraduate Medical School (London) England	NR	4	Peritonitis from perforated diverticulitis	Drainage - Drainage and colostomy - Exteriorization of the affected loop - Hartmann’ procedure - Resection with anastomosis
Botsford 1971	Harvad Medical School Massachusetts (Boston) USA	1950–70	4	Perforated diverticulitis	Drainage and colostomy - Mikulicz’ procedure – Hartmann’ procedure
Byrne 1971	Boston City Hospital (Boston) USA	NR	4	Peritonitis from perforated diverticulitis	Drainage and colostomy – Hartmann’ procedure
Miller 1971	Roosvelt Hospital (New York) USA	1957–69	4	Peritonitis from perforated diverticulitis	Drainage and colostomy – Hartmann’ procedure
Watkins 1971	Washington Medical School, (Washington) USA	NR	4	Peritonitis from perforated diverticulitis	Exteriorization of the affected loop
Whelan 1971	Saint Vincent Hospita, Worchester. USA	1956–70	4	Peritonitis from perforated diverticulitis	Drainage and colostomy – Hartmann’ procedure
Endrey-Walder 1973	Mayo Clinic (Rochester)	1961–70	4	Peritonitis from perforated diverticulitis	Drainage and colostomy – Resection and anastomosis with or without covering stoma
Labow 1973	Muhlemberg Hospital, (Painfield) USA	NR	4	Peritonitis from perforated diverticulitis	Drainage and colostomy - Hartmann’ procedure
Graves 1973	Vanderbilt University, (Nashville) USA	NR	4	Peritonitis from perforated diverticulitis	Drainage and colostomy - Hartmann’ procedure - Resection and anastomosis
Laimon 1974	University of British Columbia, (Vancouver) Canada	NR	5	Peritonitis from perforated diverticulitis	Hartmann’ procedure
Tagart 1974	Newmarket Community Hospital (Newmarket) England	NR	5	Peritonitis from perforated diverticulitis	Mikulicz’ procedure - Hartmann’ procedure
Rodkey 1974	Massachusetts General Hospital (Boston)	1964–73	4	Peritonitis from perforated diverticulitis	Drainage and colostomy - Hartmann’ procedure
Ryan 1974	St Vincent Hospital (Melburne) Australia	NR	4	Peritonitis from perforated diverticulitis	Drainage - Drainage and colostomy - Mikulicz’ procedure - Hartmann’ procedure - Resection with anastomosis
Tolins 1975	Albert Einstein College of Medicine, New York	196873	4	Perforated diverticulitis	Drainage and colostomy - Hartmann’ procedure
Nilsson 1976	Centralsarettet, (Halmastated) Sweden	1963–72	4	Peritonitis from perforated diverticulitis	Drainage - Drainage and colostomy - Mikulicz’ procedure - Hartmann’ procedure - Resection with anastomosis
Berardi 1976	Veerans Administration Hospital (Des Moines) USA	1969–73	5	Peritonitis from perforated diverticulitis	Drainage and colostomy
Saegesser 1975	Bern Switzerland	NR	5	Peritonitis from perforated diverticulitis	Mikulicz’ procedure
Classen 1976	Union Memorial Hospital (Baltimore) USA	1965–75	4	Perforated diverticulitis	Drainage and colostomy - Exteriorization of the affected loop
Himal 1977	McGill University (Montreal), Canada	NR	4	Perforated diverticulitis	Drainage and colostomy - Exteriorization of the affected loop - Hartmann’ procedure
Eng 1977	New York School of Medicine (New York), USA	1971–75	4	Peritonitis from perforated diverticulitis	Hartmann’ procedure
Nahrwold 1977	Milton S. Hershey Medical Center, (Hershey), USA	NR	4	Perforated diverticulitis	Hartmann’ procedure
Sweatman 1977	Birmingham (USA)	1962–72	4	Peritonitis from perforated diverticulitis	Drainage and colostomy - Hartmann’ procedure - Resection with anastomosis
Hinckey 1978	Montreal General Hospital (Montreal) Canada	NR	4	Peritonitis from perforated diverticulitis	Drainage and colostomy - Hartmann’ procedure
Malafosee 1978	Hopital Saint-Antomine (Paris) France	1964–74	4	Peritonitis from diverticulitis	Drainage and colostomy - Hartmann’ procedure - Exteriorization of the affected loop - Resection with anastomosis
Morgenstern 1979	Cedars-Sinai Medical Centers (Los Angeles) USA	1965–78	4	Perforated diverticulitis	Drainage and colostomy
Howe 1979	University of Arkansas (Little Rock) USA	1967–77	4	Perforated diverticulitis	Drainage and colostomy - Hartmann’ procedure
Nunes 1979	Spokane (USA)	1971–78	4	Peritonitis from perforated diverticulitis	Hartmann’ procedure
Haglund 1979	University of Gotheborg, Gotheborg, Sweden	1962–73	4	Peritonitis from perforated diverticulitis	Drainage and colostomy - Hartmann’ procedure - Exteriorization of the affected loop - Resection and anastomosis with a covering stoma
Thow 1980	Cale Clinic (Urbana) USA	1971–76	4	Peritonitis from perforated diverticulitis	Resection with anastomosis
Theile 1980	Princess Alexandra Hospital (Brisbane) Australia	NR	4	Peritonitis from perforated diverticulitis	Drainage - Drainage and colostomy - Hartmann’ procedure
Greif 1980	Beth Israel Medical Center (Boston) USA	NR	5	Peritonitis from perforated diverticulitis	Mikulicz’ procedure

**Table 5:** Conclusion of studies reported the use of peritoneal lavage and drainage, published up to 1980

Author	Technique	Quote from publication
Lockart-Mummery 1910	The toilette of the peritoneum and abdominal drainage was the only technique performed. The visible colonic perforation, if possible to find, can be closed by suture	“Perforation and general peritonitis. In these cases, though a careful toilet of the peritoneum and the establishment of adequate drainage may suffice, it is advisable, if possible to find, and close by suture, the perforation of colon”
Judd and Pollock 1924	The toilette of the peritoneum and abdominal drainage was the only technique performed. The visible colonic perforation, if possible to find, can be closed by suture	“We have operated on a number of patients who had abscesses, either just draining the abscess, or draining and suturing the opening left in the colon at the point of perforation of the diverticulum”
Eggers 1931	A taylor surgery. In the arsenal of the surgeon there is also the toilette of the peritoneum and abdominal drainage.	“In four patients an acute perforation took place into the- free- peritoneal cavity. One of them was drained early and recovered, another was treated expectantly for peritonitis without knowledge at that time of the underlying cause, and finally recovered, while the other two died”
Eggers 1941	A taylor surgery. In the arsenal of the surgeon there is also the toilette of the peritoneum and abdominal drainage	“Drainage only”
Smithwick 1942	A taylor surgery. In the arsenal of the surgeon there is also the toilette of the peritoneum and abdominal drainage. The visible colonic perforation, if possible to find, can be closed by suture	“Acute perforation. Principally ± drainage suture”
Hughes 1952	A taylor surgery. In the arsenal of the surgeon there is also the toilette of the peritoneum and abdominal drainage	“Laparotomy and simple drainage of the abdomen”
Edwards 1954	A taylor surgery. In the arsenal of the surgeon there is also the toilette of the peritoneum and abdominal drainage. The visible colonic perforation, if possible to find, can be closed by suture	“The prognosis after early operation in patients with no previous history, or a history of short duration, is excellent, for the bowel wall is still flexible and the perforation can readily be found and easily closed. The closure is reinforced by omentum and the pelvis drained. The real problem is in the surgical management of those cases in which there has been a long history of recurrent attacks of diverticulitis and in which at exploration the bowel is found to be immensely thickened and congested, and particularly in those in whom the actual point of perforation cannot be identified. In such an event the safest procedure is to exteriorize the bowel, if this is practicable. An alternative is to attempt to seal off the inflamed area with pericolic fat and omentum; in the old and the very ill patient the operation may need to be restricted to this procedure”.
Bacon 1956	A taylor surgery. In the arsenal of the surgeon there is also the toilette of the peritoneum and abdominal drainage. The visible colonic perforation, if possible to find, can be closed by suture	“Once the diverticulum rupture is discovered and the surround bowel wall is to be fairly normal it can be quickly repaired”
Ryan 1958	A taylor surgery. In the arsenal of the surgeon there is also the toilette of the peritoneum and abdominal drainage.	“Drainage alone was carried out”
Brown 1960	A taylor surgery. In the arsenal of the surgeon there is also the toilette of the peritoneum and abdominal drainage. The visible colonic perforation, if possible to find, can be closed by suture	“Laparotomy and peritoneal drainage was the operation most commonly performed as palliative measure. Identification and suture of a ruptured diverticulum in the distal colon is usually impossible owing to the friable state of tissues involved, but on occasion it can be achieved”
Greig 1960	A taylor surgery. In the arsenal of the surgeon there is also the toilette of the peritoneum and the abdominal drainage.	“Laparatomy and drainage only”

In the same decade Mikulicz described his two-stage technique of intestinal resection and anastomosis in a well-known article entitled “Surgical Experiences with Intestinal Carcinoma” presented for the first time to the Thirty-First Congress of the German Society of Surgery in 1903 [114]. Along with the description of details of his technique for intestinal resection, he also reported his personal experience in 106 patients, 16 of whom underwent a two-stage technique because he considered performing the initial anastomosis to be too hazardous for the treatment of intestinal cancer and so advocating to limiting the procedure in some cases to the resection and a double-barreled colostomy. Mikulicz strongly recommended this two-stage technique for all resections and anastomoses of the large and small bowel when the bowel was obstructed. This technique was then subsequently adopted for the treatment of diverticulitis.

In 1924 an observational study from the Mayo Clinic advocated drainage and suture of the colonic perforation as well as selective use of a diverting colostomy [115]. However, in some cases, fecal fistulas developed and some became chronic. In cases of substantial infection in or close to the colon, or in the presence of a colovesical fistula or fistula with other structures, the mortality decreased substantially with use of a diverting colostomy. However, in some cases the local suture of the perforation had been unsatisfactory because of difficulties visualizing the perforation, as well as difficulty in suturing edematous bowel wall. During the same decade Henri Hartmann proposed his surgical technique consisting of sigmoid resection, burying the rectal stump and performing terminal colostomy for the treatment of rectal cancer, as an alternative to abdomino-perineal resection, commonly called Hartmann’s procedure (HP) [116]. Hartmann had not originally advocated subsequent restoration of intestinal continuity.

In 1930 Rankin and Brown standardized the three-stage procedure developed by Mayo in 1907 [21, 115]. The first stage of the procedure consisted of peritoneal lavage, drainage of any abscess and creation of a proximal colostomy. The second stage was performed after a period of 2 to 4 months and involved resection of the sigmoid colon with end-to-end anastomosis. The third stage consisted of closure of the colostomy a few weeks after the second stage to ensure healing of the anastomosis.

In 1934 Lockhart-Mummery changed his original surgical technique, based only on peritoneal toilette and abdominal drainage by adding the use of a proximal diverting colostomy [23]. The right half of the transverse colon was used to create the colostomy. This approach was subsequently shown to leave the left colon and splenic flexure free from adhesions and favor the ensuing sigmoidectomy [117]. However, drainage and colostomy were associated with a higher mortality and morbidity because further leakage occurred from the site of perforation in spite of the proximal colostomy in some cases. Moreover, the inflamed colon could represent a source of sepsis and leaving this *in situ* was not seen as an attractive option. For these reasons, many surgeons, including Arhneim and Egger, favored the Mikulicz procedure [24, 118]. However, while most surgeons approved the theoretical advantages of Mikulicz’ exteriorization, they believed that this technique could be performed in very limited cases, because of the surrounding adhesions and edematous bowel [25, 119].

The three-stage procedure was the standard treatment of acute diverticulitis until the late 1940’s. “Palliative” treatment, consisting of formation of a colostomy alone, without subsequent resection, was almost partially abandoned during this period. Interval closure of the colostomy without colon resection led to a high proportion of aggravation of symptoms– over 45 and 70% of patients, in Smithwick’s and Pemberton*et al.*’s experiences, respectively [27, 28]. During this period, the use of the HP for the treatment of recto-sigmoid cancer decreased in favor of resection and primary anastomosis (PRA). At the same time, HP gained in popularity as a treatment of complications of DD and other emergency conditions while antibiotics were introduced into clinical practice. In 1950 Boyden articulated this approach and proposed a technical variation: a long distal bowel stump was exteriorized through the hypogastrium [29].

During this period, drainage of pus and formation of a proximal colostomy with the aim of controlling severe sepsis was not longer popular: fecal diversion was thought to limit peritonitis and surgeons avoided source control in fear of spreading infection. In the 50’s such ‘palliative’ operations were increasingly considered as unsatisfactory. The resection of the inflamed colonic segment followed by an anastomosis was suggested as a more ‘bold’ alternative. The improvement of anesthetic techniques and antibiotic therapy supported this approach of “eliminating the source of sepsis”, as stated by Crile in 1954 or “source control” as later coined by Marshall [120, 121]. It seems that a direct approach to the source of contamination, with diversion or resection of diseased segments, drainage of abscess, and suction of most of the pus and feces from abdominal cavity, gave the patient the best chance of survival [120]. Also during this period PRA with or without proximal protective colostomy was increasingly reported [32–35]. The results were good in the presence of a minimum peri-diverticular contamination or intra-mesenteric abscess [34].

The gold standard surgical treatment of complicated diverticulitis was the three-stage procedure with the drainage and colostomy as first stage. Stauton and Smiley supported the diversion of the perforated loop with sigmoid colostomy in the left iliac fossa plus drainage and colostomy [49, 50, 59–61]. Transverse colostomy was discouraged for fecal peritonitis as it might leave fecal residue proximal to the perforation [122]. Certainly the septic focus was removed from the peritoneal cavity, but the toxins were not removed from the circulation and still exerted their systemic effect [123, 124].

During the 1970s HP gained renewed popularity, because eradicating the source of sepsis had proved its superiority to mere diversion in terms of mortality and complications. Subsequent colostomy closure became an increasingly routine procedure, well standardized and progressively feasible [5, 84–113].

In the 1980s a landmark systematic review by Krukowski and Matheson was published [125]. The two authors examined the mortality in 36 case series (821 cases of diverticulitis associated with purulent or fecal peritonitis) published between 1957 and 1984 that compared resection versus colostomy without resection. All patients underwent emergency surgery: mortality was 12% in the 316 who underwent resection versus 29% in the 505 who underwent colostomy without resection. Although there was a high risk of selection bias (patients in better health were more likely to undergo resection while patients with poor health status were more likely to receive a colostomy), this report found that, with antibiotics and better fluid resuscitation therapy, a substantial number of patients could undergo resection as an emergency procedure with an acceptable mortality rate. In addition, advocates of resection argued that emergency colectomy avoided the risk of missing colonic cancer (which was estimated to occur in 2–7% of the cases) and decreased morbidity [125].

The 1990s saw the emergence of two-stage resection and HP supported by two RCTs published in Denmark and France [126, 127]. Kronborg, in a single-center study published in 1993, examined 62 patients operated on for diverticulitis complicated with peritonitis, 46 of whom were classified as Hinchey III (purulent peritonitis). Twenty-one were randomized to transverse colostomy, suture and omentoplasty without resection with 100% survival. Six of the 25 patients (24%) randomized to acute resection without primary anastomosis died post-operatively [126]. Kronborg concluded that simple suture of the perforation and omentoplasty with proximal diversion was safer and more effective than acute resection in purulent peritonitis and comparable in effectiveness [126]. Hospital stay was shorter and there were fewer inflammatory relapses after acute resection. Twenty-seven different surgeons operated on the 62 enrolled patients during 14 years. This RCT was suspended early due to slow recruitment (an average of four patients each year) and used subgroup analysis without statistical adjustment, and consequently lacked statistical power [126]. In contrast, the French multicenter prospective RCT included 103 patients with either purulent (Hinchey III) or fecal peritonitis (Hinchey IV) [127]. The primary endpoint was post-operative peritonitis. For the 48 patients who were randomized to colostomy (with perforation closure by suture in the Hinchey IV cases), the postoperative peritonitis rate was high, up to 20%. In contrast, for the other 55 patients randomized to HP emergency resection, the postoperative peritonitis rate was significantly lower, less than 2%. Three studies were published from 1985 to 2000 where the HP was compared to the three-stage technique (Table 6) [126, 128, 129]. Our recent meta-analysis of these three studies analyzed the overall mortality as the primary outcome [130]. A total of 159 patients had colonic resection versus 105 who maintained perforated diseased segment of colon after proximal diversion or suture of the colon perforation. Overall, mortality was 13.6% (20/147) in the colonic resection group versus 24.6% (18/73) after proximal diversion or suture of the colon perforation (with perforated diseased segment of colon maintained). Statistical analysis failed to show a statistically significant lower overall perioperative mortality rate in the colonic resection group compared to the other group (OR 0.53, 95% CI 0.16 to 0.73, *P =* 0.31) and heterogeneity among the included studies was high (Tau^2^ = 0.71, Chi^2^ = 5.31, I^2^ = 62%) [130]. In 2000, the American Society of Colon and Rectal Surgeons based on expert review of the evidence concluded that segmental colonic resection followed by an end colostomy (i.e., HP) was the most suitable procedure for perforated diverticulitis with peritonitis [131].

**Table 6:** Hartman procedure vs to three stages technique

	Study type	Cases		Age (yr)	Pathology	Hinchey stage			
						Hinchey </= 2		Hinchey > 2	
		Resection	Trasverse colostomy and drainage			resection	colostomy and drainage	resection	colostomy and drainage
Nagorney et al. (1985)	R	90	31	CR 61 vs TCD 65	P	−	−	90	31
Finlay et al. (1987)	R	38	40	D	P, A	12	29	26	11
Kronborg et al. (1993)	RCT	31	31	CR 73 vs TCD 71	P	−	−	31	31

*RCT* randomized controlled trial, *R* retrospective observational trial

*CR* colon resection, *TDC* transverse colostomy and drainage

Pathology: *P* peritonitis, *A* abscess; *O* obstruction, *DD* diverticular disease

During this period a number of systematic reviews and meta-analysis were published comparing PRA versus HP (Table 7) [130, 132–135].

**Table 7:** Systematic review: primary resection with anastomosis vs Hartmann’s procedure

Authors	Type of review	Number of studies included	Number of patients included	Conclusion
Salem 2004	systematic review	98	1.051	“Reported mortality and morbidity in patients with diverticular peritonitis who underwent primary anastomosis were not higher than those in patients undergoing Hartmann’s procedure were. This suggests that primary anastomosis is a safe operative alternative in certain patients with peritonitis. Despite inclusion of only patients with peritonitis in this analysis, selection bias may have been a limitation and a prospective, randomized trial is recommended.”
Constantinides 2006	systematic review and metanalysis	15	963	“Patients selected for primary resection and anastomosis have a lower mortality than those treated by Hartmann’s procedure in the emergency setting and comparable mortality under conditions of generalized peritonitis (Hinchey > 2). The retrospective nature of the included studies allows for a considerable degree of selection bias that limits robust and clinically sound conclusions. This analysis highlights the need for high-quality randomized trials comparing the two techniques
Abbas 2007	systematic review	18	884	“This review suggests that surgical resection and primary anastomosis in acute diverticulitis with peritonitis compares favourably with Hartmann’s procedure in terms of peri-operative complications. The need for revision of Hartmann’s procedure could be subsequently avoided. Some articles showed that patients with severe peritonitis, who had a diverting stoma, in the setting of resection and primary anastomosis, had the lowest complication rate. However, the quality of these studies was poor with the presence of selection bias.”
Cirocchi 2013	systematic review and metanalysis	14	1041	*“Despite numerous published articles on operative treatments for patients with generalized peritonitis from perforated diverticulitis, we found a marked heterogeneity between included studies limiting the possibility to summarize in a meta-analytical method the data provided and make difficult to synthesize data in a quantitative fashion. The advantages in the group of colon resection with primary anastomosis in terms of lower mortality rate and postoperative stay should be interpreted with caution because of several limitations. Future randomized controlled trials are needed to further evaluate different surgical treatments for patients with generalized peritonitis from perforated diverticulitis.”*
Lorusso 2016	systematic review and metanalysis	24	4.062	“*Our meta-analysis shows that the PRA technique is better than HP for all considered outcomes. Due to the high variability of the included studies, further randomized controlled trials would be required to confirm these results”.*

The first published was Salem’s review in 2004 [132]. This review identified 98 studies (published between 1957 and 2003) on the surgical management of perforated diverticulitis complicated with peritonitis. Perioperative mortality data from patients with diverticular peritonitis undergoing HP (*n =* 1,051) was calculated for 54 studies. Overall cumulative mortality rate was 19.6% (18.8% for HP and 0.8% for subsequent procedures to restore intestinal continuity). The surgical site infection rate was 29.1% (24.2% for HP and 4.9% for reversal). Stoma complications and anastomotic leaks (after restoring intestinal continuity) occurred in 10.3% and 4.3%, respectively. Of 569 reported cases of PRA from 50 studies, the associated mortality rate was 9.9% (range 0–75%) with an anastomotic leak rate of 13.9% (range, 0–60%) and a surgical site infection rate of 9.6% (range, 0–26). In patients with diverticular peritonitis who underwent PRA the reported mortality and morbidity rates were not higher than those in patients undergoing HP, suggesting that PRA was a safe operative option in this specific population.

However, in 2006, Constantinides et al. published a systematic review of 15 observational studies (13 retrospectives and 2 prospective nonrandomized studies published from 1984 to 2004) comparing PRA with HP for acute diverticulitis in emergency surgery [133]. They found that peri-operative mortality was lower in those patients who underwent PRA compared with those who underwent HP. In addition, there was a trend favoring PRA for surgical complications (surgical site infections, abscesses, and peritonitis). However, it has to be borne in mind that this review was at high risk of selection bias because of the primarily retrospective character of case series Notwithstanding, these data showed that: 1) emergency PRA could be performed in selected cases with a low incidence of anastomotic leak (roughly 6%); 2) PRA and HP had similar operative times; and 3) for the most severe cases (Hinchey IV), PRA and the HP had similar mortality (14.1 vs. 14.4%).

In 2006, the American Society of Colon and Rectal Surgeons updated their guidelines for the treatment of sigmoid diverticulitis dating from 2000 [136]. Emergency sigmoid resection was deemed mandatory for diverticulitis with peritonitis, and the alternatives to the HP consisted of PRA with or without a de-functioning stoma. The role of the PRA (particularly without the use of a de-functioning stoma) remained unclear.

In 2007, Abbas published a systematic review of trials conducted between 1966 and 2003 [134]. Eighteen non-randomized studies reporting on 884 matched patients with complicated diverticulitis were included. There were no significant differences found between PRA and HP in terms of mortality, morbidity, sepsis, surgical site complications, duration of operation or antibiotic therapy. However, again the risk of selection bias was high.

Successively a RCT comparing PRA and HP was published [137]. The study protocol called for a de-functioning ileostomy within the PRA procedure. Ninety patients were randomized to PRA or HP in 14 centres in eight countries during a 9-year period [137]. Thirty-four PRA patients were compared to 56 HP patients. There were no statistically significant differences found in terms of age (*P =* 0.481), gender (*P =* 0.190), Hinchey stage III and IV (*P =* 0.394) and Mannheim Peritonitis Index (*P =* 0.145). There were no statistically significant differences found in mortality (2.9 vs. 10.7%; *P =* 0.247) or morbidity (35.3 vs. 46.4%; *P =* 0.38) after PRA or HP. The rate of restoration of intestinal continuity was similar in both groups (64.7% after PRA and 60% after HP, *P =* 0.659) after a similar lag time between emergency operation and elective stoma reversal (*P =* 0.43). The main difference between the two groups was the post-operative complication rate after restoration of intestinal continuity that differed statistically significantly (4.5 vs. 23.5% after PRA and HP, respectively; *P =* 0.05). However, it is impossible to draw firm conclusions from this RCT because of early termination and enrolment of only 15% of its calculated sample size (600 patients to achieve 90% power to detect 10% difference in mortality).

Another systematic review and meta-analysis on the same topic, published in 2013, compared PRA and HP [130] for the treatment of diverticulitis complicated by peritonitis, including the above mentioned RCT [137]. The overall morbidity rate was 17% (40/235) and 28.37% (84/296) in the PRA and HP groups, respectively (OR 0.46, 95% CI 0.23 to 0.90, *P =* 0.02). The re-intervention rate after PRA did not differ significantly between the two groups (15.2% versus 24.1% in the PRA and HP, respectively; OR 1.06, 95% CI 0.24 to 4.73, *P =* 0.94). Successively another RCT was published on this topic; Oberkofler and colleagues randomized 62 patients in four centres with acute perforation of left colon (Hinchey III and IV) to HP (*n =* 30) or PRA (with de functioning ileostomy, *n =* 32) with a planned procedure to restore intestinal continuity after 3 months in both groups [138]. Both groups were similar at baseline (Hinchey III: 76% vs. 75% and Hinchey IV: 24% vs. 25%, for HP vs. PRA respectively). The primary outcome was the overall complication rate that was similar in both groups (80% vs. 84%, *P =* 0.813). The outcomes after the primary colon resection were also similar (mortality 13% vs. 9% and morbidity 67% vs. 75% in HP vs. PRA). This is the first RCT that seems to favour PRA in patients with complicated diverticulitis with peritonitis. However, there is evidence of bias, as highlighted by Panis in his comments, therefore no firm conclusions can be drawn [139]. Succesively Lorusso published a systematic review including 24 studies, in [135] which reported the same our results (Table 8) [137, 138, 140–162].

**Table 8:** Evidence about primary resection with anastomosis vs Hartmann’s procedure

	Study type	Cases		Pathology	Hinchey stage			
					Hinchey </= 2		Hinchey > 2	
		PRA	HP		PRA	HP	PRA	HP
Hold et al. (1990)	R	99	76	DD, P, A	83	45	16	31
Gooszen et al. (2001)	R	32	28	DD, P, A	11	9	21	19
Schilling et al. (2001)	PNR	13	42	DD, P	0	0	13	42
Regenet et al. (2003)	PNR	27	33	DD, P	0	0	27	33
Richter et. Al (2006)	PNR	36	5	DD, P	0	0	36	5
Trenti et al. (2011)	R	27	60	P	0	0	58	69
Oberkofler et al. (2012)	RCT	32	30	(DD) P	0	0	32	30
Alanis et al. (1989)	R	34	26	DD,P,A	31	19	3	7
Alizai (2013)	R	26	72	DD,P,A	16	24	10	48
Blair (2002)	R	33	634	DD,P,A	24	31	9	32
Berry (1989)	R	27	47	DD,P,A,O,F,B	NR	NR	NR	NR
Gawlick (2012)	R	340	1678	DD,P,A	NR	NR	NR	NR
Herzog (2011)	R	21	19	DD,P,A,O,B	NR	NR	NR	NR
Kourtesis (1988)	R	23	10	DD,P,A, F	NR	6	0	4
Mäkelä (2005)	R	64	93	DD,P	62	19	2	0
Mueller (2011)	R	47	26	DD,P,A	45	14	2	12
Pastenak (2010)	R	46	65	DD,P,A	34	17	12	48
Saccomani (1993)	R	26	8	DD,P,A,F	NR	NR	NR	NR
Smirniotis (1992)	R	6	18	DD,P,A	6	10	0	8
Stumpf (2007)	R	36	30	DD,P,A,O	NR	NR	NR	NR
Tabbara (2010)	R	18	176	DD,P,A,S	16	69	2	107
Zingg et al. (2009)	PNR	46	65	DD, P	34	17	12	48
Binda et al. (2012)	RCT	34	56	P	0	0	34	56
Tudor (1994)	PNR	76	77	DD,P,A,B,O,F	29	20	8	44
Vermeulen (2007)	R	61	139	DD,P,A	35	44	26	95

*RCT* randomized controlled trial, *PNR* prospective, nonrandomized, *R* retrospective

*PRA* primary resection and anastomosis, *HP* Hartmann’s operation

Pathology: *P* peritonitis, *A* abscess, *O* obstruction, *DD* diverticular disease

*F* fistula, *B* bleeding (dc chronic diverticulitis)

Recently laparoscopic peritoneal lavage (LPL) with drainage and antibiotics has been recently introduced into the surgical practice with aim to decrease the rate of HP [163, 164]. In 2009, Toorenvliet’s systematic review identified 231 patients with acute diverticulitis who underwent LPL, drainage and antibiotics therapy [165]. In 95.7% of patients this minimally invasive procedure permitted adequate control of the abdominal and systemic sepsis, with low rates of mortality (1.7%), morbidity (10.4%) and stoma (1.7%). Most patients subsequently had a delayed elective laparoscopic PRA. Patients who did not undergo subsequent resection had a long recurrence free period. The authors concluded that LPL was an effective and safe treatment of peritonitis secondary to perforated diverticulitis [165].

However, the use of peritoneal lavage without primary resection in generalized peritonitis originating from perforated diverticulitis remains controversial. Recently three RCT (DILALA-trial, SCANDIV-trial, LADIES trial) including a total of 343 participants (178 in the lavage group versus 175 in the resection group) have been published on this topic (Table 9) [166–168].

**Table 9:** RCTs about abdominal laparoscopic lavage: characteristics

Name of trial Trial registry entries	Type of trial	Country	Participants	Inclusion criteria	Exclusion criteria	Study number	Time of study
LADIES ClinicalTrials.gov Identifier: NCT01317485	Multicentre two-armed randomised trial: 34 teaching hospitals and eight academic hospitals in Belgium, Italy, and the Netherlands	The Netherlands	Patients with generalised purulent and faecal peritonitis from sigmoid diverticulitis	Clinical signs of peritonitis. Free gas on and/or diffuse fluid on CT LOLA arm: Only patients with purulent perforated diverticulitis without overt perforation	Dementia Previous sigmoidectomy Prior pelvic irradiation, Chronic treatment with high-dose steroids (>20 mg daily) Being aged younger than 18 years or older than 85 years Preoperative shock needing inotropic support Patients with Hinchey I and II Patients with Hinchey IV peritonitis or overt perforation were excluded from the DIVA group	LOLA arm: 264 DIVA arm: 212	LOLA arm: between July 2010, and the early termination of the trial February 2013
DILALA trial ISRCTN for clinical trials ISRCTN82208287	Multicentre randomised trial	Sweden- Denmark	Perforated non-faeculent diverticulitis	Hinchey grade III at diagnostic laparoscopy, i.e. free fluid	Hinchey grade I - II at laparoscopy i.e. no free fluid Hinchey grade IV at laparoscopy, i.e. gross faecal contamination. Other pathology than diverticulitis diagnosed as explanation of peritonitis	80	Between February 2010 until February 2014
SCANDIV ClinicalTrials.gov Identifier: NCT01047462	Multicentre randomised trial	Sweden- Norvey	Perforated non-faeculent diverticulitis	Patients with generalised peritonitis	Pregnancy Bowel obstruction	199	Between February 2010 until June 2014

The DILALA trial included patients with only Hinchey III peritonitis diagnosed by laparoscopy and with 1-year re-operation rate as primary outcome. The preliminary analysis of the short-term results (12 weeks) in 76 patients reported no statistically significant difference regarding morbidity and mortality, statistically significant longer period of abdominal drainage but shorter hospital stay in the LPL group compared to HP group [167].

LADIES was a two-arm trial with 1-year morbidity and mortality as the primary outcome. The LOLA arm compared laparoscopic lavage with sigmoidectomy in 90 patients with Hinchey III diverticulitis [166]. The trial could not prove the superiority of LPL and was terminated due to increased adverse events in this group despite the lack of statistical significance.

In contrast, the SCANDIV trial was able to report on the totality of 199 patients randomized to laparoscopic lavage or to laparoscopic/open resection with or without anastomosis [168]. The primary outcome was 90-day major complications rate according to the Clavien-Dindo classification. The authors reported a non-statistically significant higher incidence of the primary outcome in the LPL group and comparable mortality. However, there were statistically significantly higher rate of abscesses, secondary peritonitis and re-operations and in the LPL group along with missed malignancy in four cases. Despite the shorter operative time in the LPL group (72 vs 149 min), the hospital stay was similar in both groups. However, the study has several limitations such as inclusion of patients with Hinchey I and II and participation of more experienced surgeons in the resection group, which might be a source of significant bias [169].

Slim recently published a letter in which the three RCTs were examined and none showed laparoscopic lavage to be superior. In relation to three meta-analysis of these studies could respond to the question raised by this editorial, but “… came to opposite conclusions…..” [170].

We published the fourth meta-analysis, that failed to demonstrate significant benefits (Table 10) [171–174]. Overall the quality of evidence was low because of serious concern regarding the risk of bias and imprecisions. A significantly increased rate of intra-abdominal abscess formation (RR = 2.54, 95% CI 1.34 to 4.83) (moderate quality of evidence), was seen with this approach. However, LPL does not appear inferior to traditional surgical resection and may achieve reasonable outcomes (lower rate of post-operative wound infections, R = 0.10, 95% CI 0.02 to 0.51) and less hospital resources (shorter duration of post-operative hospital stay during index admission, WMD =−2.03, 95% CI −2.59 to −1.47).

**Table 10:** Meta-analysis about laparoscopic abdominal lavage

	Cirocchi		Ceresoli	Angenete	Marshall
	All Hinchey	Hinchey III	Hinchey III	Hinchey III	Hinchey III
Post-operative mortality at index admission or within 30 days from index intervention	RR 1.33, 95% CI 0.37 to 4.74	RR 3.01, 95% CI 0.48 to 18.93	OR 0.93; 95% C.I. 0.23–3.82; *P =* 0.92	RR 1.34, 95% CI 0.59–3.04	RR 1.34, 95% CI 0.37 to 4.79
Mortality at 90 days	RR 1.27, 95% CI 0.60 to 2.69	Not performed^j^	OR 0.83; 95% C.I. 0.32–2.11; *P =* 0.69	RR 0.86, 95% CI 0.40–1.83	RR 0.86, 95% CI 0.40 to 1.84
Mortality at 12 months	RR 0.84, 95% CI 0.38 to 1.88	Not performed^j^	OR 0.74 *P =* 0.51	RR 0.54, 95% CI 0.38–0.76	
Reoperation at index admission or within 30 days from index intervention	RR 1.93, 95% CI 0.71 to 5.22	RR = 1.40, 95% CI 0.71 to 4.90	OR 3.75, *P =* 0.006	RR 1.34, 95% CI 0.59–3.04	RR 3.03, 95% CI 1.16 to 7.89
At 90 days follow reoperations	Not analyzed^a^	Not performed^j^	NR	RR 1.71, 95% CI 0.85–3.43	NR
At 12 months follow reoperations	RR 0.57, 95% CI 0.39 to 0.86	Not performed^j^	OR 0.32, *P =* 0.0004	RR 0.54, 95% CI 0.38–0.76	NR
Intra-abdominal abscesses at index admission or within 30 days from index intervention	Not analyzed^b^	Not performed^j^	OR 3.50; 95% C.I. 1.79–6.86; *P =* 0.0003	NR	NR
Intra-abdominal abscesses at 90 days	RR = 2.54, 95% CI 1.34 to 4.83	Not performed^j^	NR	NR	NR
Wound infections	RR = 0.10, 95% CI 0.02 to 0.51	Not performed^j^	OR 0.14; 95% C.I. 0.04–0.45; *P =* 0.0009	NR	NR
Morbidity at 90 days	Not performed^j^	Not performed^j^	OR 1.70; 95% C.I. 1.00–2.87; *P =* 0.05	NR	NR
Presence of stoma at 12 months	RR = 0.50, 95% CI 0.14 to 1.75	Not performed^j^	OR 0.44 *P =* 0.27	NR	RR 0.50, 95% CI 0.14 to 1.76
Operating time	Not analyzed^c^	Not performed^j^	NR	NR	NR
Post-operative persistent peritonitis	Not analyzed^d^	Not performed^j^	NR	NR	NR
Post-operative secondary peritonitis	Not analyzed^e^	Not performed^j^	NR	NR	NR
Length of post-operative hospital stay during index admission.	WMD −2.03, 95% CI−2.59 to−1.47	Not performed^j^	NR	NR	NR
Adverse events within 90 days by Dindo-Clavien grade I-II	Not analyzed^f^	Not performed^j^	NR	NR	NR
Adverse events within 90 days by Dindo-Clavien grade IIIa	Not analyzed^g^	Not performed^j^	NR	NR	NR
Adverse events within 90 days by Dindo-Clavien grade IIIb	RR 1.40, 95% CI 0.47 to 4.17	Not performed^j^	NR	RR 1.46, 95% CI 0.99–2.20	NR
Adverse events within 90 days by Dindo-Clavien grade IVa	RR 0.59, 95% CI 0.20 to 1.75	Not performed^j^	NR	NR	NR
Adverse events within 90 days by Dindo-Clavien grade IVb	RR 0.62, 95% CI 0.10 to 3.75	Not performed^j^	NR	NR	NR
Total length of hospital stays within 12 months.	Not analyzed^h^	Not performed^j^	NR	NR	NR
Quality of Life	Not analyzed^i^	Not performed^j^	NR	NR	NR
Post-operative ICU admission	NR	NR	NR	NR	RR 0.85, 95% CI 0.40 to 1.78

*NR* not reported

^a^Two trials (LADIES and DILALA) reported this outcome, but the LADIES reported only the abscesses needing percutaneous drainage, differently the DILALA reported only the abscess underwent surgical reintervention

^b^Only one trial (SCANDIV) reported this outcome

^c^This outcome was reported in the SCANDIV although a definition of operating time was not provided. In the DILALA trial, the duration of surgery, and time between end of surgery and the end of anesthesia was reported. In the LADIES trial, the results of a comparative analysis were provided in the absence of the primary data. Because of the incongruous reports of operating time, for these reasons the analysis of this outcome was not performed

^d^Only the DILALA trial analyzed this outcome as persistent peritonitis

^e^Only the SCANDIV trial analyzed this outcome of persistent peritonitis

^f^Only the DILALA trial reported this outcome

^g^Only the DILALA trial reported this outcome

^h^Only the DILALA trial reported this outcome

^i^All the included trials reported this outcome; however, the data of quality of life questionnaires were not comparable

^j^Lack of data

## Discussion

This review analyzes the best scientific evidence for over a century of surgery for complicated DD stratifying the technical solutions over time.

It is difficult to draw firm conclusions based on the available evidence. The studies were principally retrospective and prone to bias, irrespective of the decade in question. Also the few currently available RCTs have limitations and therefore have not been able to provide clear recommendations.

Since the publication of Graser in 1899, DD has been a subject of increasing interest for clinicians and surgeons [175]. DD is more frequent in Western countries (especially left-sided) and a trend toward increased frequency has been noted with estimated prevalence of 20–60% [176].

Despite the intensive research since the beginning of the last century, decisions regarding if and when to operate on patients with diverticulitis remains a topic of substantial debate. The debate between supporters of non-operative and traditional techniques has existed over this time and persists to this day. However, no single treatment strategy has dominated in terms of efficacy or safety.

Diverticulitis is complicated in approximately 10–25% of all cases. Operations are traditionally reserved for complicated diverticulitis (colon perforation and peritonitis, abscess, fistula, or stenosis). After a first acute attack of diverticulitis, approximately 20–30% of patients undergo surgery, around a half of these cases being performed as an emergency. Of these, 15–40% tend to be young patients (less than 50 years old). The mortality of emergency operations is between 10 and 20% while in elective surgery it is less than 2% [177] The condition has a great social and financial impact. In USA about 313.000 hospitalizations are due to diverticular disease with more 50.000 bowel resections annually [178, 179].

A retrospective cost analysis from USA found that treatment of DD for 1 financial year was 5.3% of the total annual budget of General Surgery [180]. At present, diverticulitis is the associated diagnosis for one third of all colostomies and/or colon resections [132].

Recent literature has reported an increase in the incidence of DD among younger patients. In a large review of the Nationwide Inpatient Sample (NIS) of 267,000 admissions for AD between 1998 and 2005, incidence rates increased dramatically in 18 to 44 year-olds and 45 to 64 year-olds, while they remained stable in 65 to 74 year-olds and actually decreased in persons 75 years of age or older [181].

Generally, more aggressive treatments have been used in patients in better health with less aggressive options reserved for patients in a poor state of health. Therefore, direct comparisons between such treatments in an observational study setting could lead to the erroneous conclusion that the more aggressive interventions are associated with lower morbidity and mortality than the conservative options.

It is interesting to note that some of the oldest described therapeutic options, such as the peritoneal lavage and drainage or surgery in several stages, are still very relevant today. This is particularly true on the background of the new potent antimicrobial agents, improved ICU management, the wider use of percutaneous drainage and last, but not least due to the growing experience with laparoscopic surgery.

Antimicrobial therapy plays an important role in the management peritonitis from complicated acute diverticulitis. Judicious use of antibiotics should be considered an integral part of good clinical practice. It can maximize the utility and therapeutic efficacy of treatment, and minimize the risks associated with emerging infections and the selection of resistant pathogens. Antimicrobial therapy is typically empiric because critically-ill patients need immediate treatment and microbiological data usually requires more than 24/36 h for the identification of pathogens and antibiotic susceptibility pattern.

In the last years several guidelines have been published in literature in the setting of intra-abdominal infections [182–186]. However, consideration of local epidemiological data and regional resistance profiles should be essential for antibiotic selection.

Considering the intestinal micro biota, patients with acute diverticulitis requires antibiotics toward gram-positive and gram-negative bacteria, as well as for anaerobes. Most of the complicated acute diverticulitis is community acquired infection. In these condition the main resistance threat in intra-abdominal infections may posed by Extended-Spectrum Beta-Lactamase (ESBL)-producing Enterobacteriaceae, which are becoming common in community-acquired infections worldwide [182]. The most significant risk factors for ESBL producing infection include prior exposure to antibiotics and co morbidities requiring concurrent antibiotic therapy [182].

The duration of therapy should be shortened as much as possible if there no signs of ongoing infections. Patients who have signs of sepsis beyond 5 to 7 days of treatment need diagnostic investigation to determine an ongoing uncontrolled source of infection [187].

In the management of critically ill patients with sepsis and septic shock clinical signs and symptoms as well as inflammatory response markers such as pro calcitonin, although debatable, may assist in guiding antibiotic treatment [187].

However, the variety of available treatments and the paucity of good quality evidence make clinical decision making difficult for surgeons especially in emergency setting.

Tracking the development of current surgical practice is very important due to several reasons. Firstly, we pay tribute to our teachers who paved the way to our current achievements. Secondly, analyzing historical procedures allows us to understand that these are still very relevant despite the technological advancements; we can still learn from them and further develop such techniques.

The treatment of complicated DD in the early decades was dominated either by only lavage with/without suture or different stage-procedures. In the 70’s HP gradually became increasingly popular and a routine procedure with acceptable mortality and morbidity, probably due to higher rate of successful colostomy closure at the second stage. The period 1991–2000 is characterized by increased frequency of the re-sectional surgery but the first two RCTs reported controversial results. From 2001 until now there has been a marked shift in surgical practice toward PRA and wider use of the laparoscopic approach with or without resection. This is probably due to the growing recognition that Hartmann’s reversal is not a benign procedure with 49-55% morbidity and 20% mortality rates [188–190]. Moreover, a large number of patients never undergo restoration (48–74%), albeit that patients with diverticular disease have significantly higher reversal rate (83%) [190, 191].

The studies published in 2001–2016 can be divided into two categories – comparing HP versus resection with PRA and those investigating the effectiveness of LPL, drainage and antibiotic therapy as an initial approach versus resection.

Several comparative studies published in this period reported improved outcome after resection with primary anastomosis in contrast to Hartmann’s procedure such as those of Salem et al., Constantinides et al., whereas the work of Abbas et al. showed similar results with respect to morbidity and mortality rates, duration of the operation and antibiotic therapy [132–134]. Two RCTs directly compared PRA and HP. Binda et al. reported no significant difference in mortality and morbidity rates, but significantly lower complication rate after intestinal continuity restoration in PRA versus HP [137]. However, it was stopped due to insufficient recruitment rate. A other trial found similar complication rate [138]. A recent systematic review [130] found that despite the growing body of the literature there is a marked heterogeneity between studies, which precludes the possibility to draw valid conclusions. PRA was associated with lower, but insignificant mortality rate and significantly shortened hospital stay. Generally, the benefit of PRA seems to be of lower mortality rate and shorter postoperative stay. The studies failed to write a definitive word on this issue, because their premature conclusion and bias, and, for many reasons (the laparoscopic lavage procedure’s widespread included), nowadays a study like this seems impractical.

The second group of studies has been dealing with the still controversial role of LPL with drainage and antibiotic therapy. Recently there has been a steady trend toward this approach as a definitive treatment or as bridge procedure to subsequent delayed resection due to the well-known advantages of the laparoscopic surgery and in order to reduce the rate of HP.

The systematic review of Toorenviliet et al. reported adequate control of the infection and successful delayed laparoscopic resection in most of the cases [165].

The more recent systematic review analyzed 19 studies on LPL with follow-up between 1.5 and 96 months. In 24% of the cases it was sufficient treatment with re-intervention rate 5% and 30-day mortality 4.8%. Re-admission rate was 7% with redo surgery in 69% of the cases of which 92% underwent PRA. In 36% two-stage laparoscopic management was performed [192].

None recent RCT trials [166–168] did show any significant advantage of LPL with respect to post-operative mortality and surgical re-interventions. The overall picture seems to be mostly of equivalence, except for higher re-operation rates in the LPL group, as seen in two out of the three trials. It is noteworthy that re-operation after LPL in the prematurely terminated LADIES trial did not result in excess mortality. The SCANDIV study was strongly criticized by some due to several limitations such as inclusion of patients with Hinchey I and II and participation of more experienced surgeons in the resection group which may be sources of significant bias [169, 193].

Furthermore in early 1980 some authors reported the routine use of ureteral catheters to minimize the incidence of ureteral injury during colorectal surgery (from 0.2 to 4.5%) [194–197]. In the following decades, with introduction of laparoscopic colectomy the prophylactic placement of ureteral catheters during colorectal surgery has been recommended for prevention of ureteral injuries [198, 199]; so some surgeons reported the use of lighted ureteral stents during colectomy [200]. In complicated diverticulitis the sigmoidectomy is a surgical challenge for the fibrotic adherences with the ureter. Moshe Schein reported that “*Severely” plastered diverticulitis has been referred to by some as “malignant diverticulitis” and claimed to be a contra-indication to resection. Using an appropriate technique and staying “near the bowel” an experienced surgeon should be able to resect any sigmoid*” [107, 201, 202]. It follow that in these complicated diverticulitis the prophylactic placement of ureteral catheters can reduce the ureteral injuries. The use of prophylactic ureteral catheters was reported in the surgical treatment of complicated diverticulitis, but the use of these catheters was performed only in election surgery and often during laparoscopic colectomy.

The last problem are the localized peritonitis, that not properly treated can evolve into an abdominal abscess, that are associated with an acute mortality of 5–10%. Treatment of these abscess depends on size, localization and patient’s general condition. Though solid supporting evidence is lacking, most abscess ≤ 3 cm in diameter are treated safely with antibiotics. For larger abscesses there is much evidence supporting the advantages of percutaneous drainage combined with antibiotics [203–205]. There is no evidence supporting a specific drainage or the aspiration technique. If an abscess cannot be drained percutaneously, an urgent surgical procedure is advised. Resection with primary anastomosis is the intervention of choice: there is increasing evidence that a drainage through a laparoscopic approach can be successful avoiding a further resection or deferiing it to an elective setting. After a successful percutaneous drainage of an abscess there is no agreement or evidence supporting a conservative or surgical policy [206].

Despite limitations due to the lack of strong evidence for the reasons discussed above, we summarize and propose a treatment for various clinical scenarios below:

- Patient in a good general condition with Hinchey I or II – Initially stabilize with medical treatment with or without percutaneous drainage; followed by elective PRA without protective stoma if required and/or suitable.
- Above scenario, but non-responder to initial management: two-stage procedure (emergency HP with or without a mucous fistula, followed by elective reversal if suitable) or PRA with or without a de functioning stoma.
- Selected cases Hinchey II-III – LPL.
- Hinchey III not suitable for LPL – PRA or HP.
- Hinchey IV – HP.

On this issue the results of two ongoing studies are waited: one RCT study, LapLand and one multicenter retrospective study on patients submitted to laparoscopic lavage, the LLOStudy [207, 208].

After preliminary promising results [209], future ongoing experiences might confirm the feasibility and demonstrate the safety of laparoscopy and primary anastomosis even in cases of selected, hemodynamically stable, patients with Hinchey IV perforated diverticulitis and feacal peritonitis, if performed by experienced colorectal surgeons [210].

## Conclusion

The management of patients with colon perforation from diverticulitis is still evolving. During the late 19th century initial lavage with or without simple suture and drainage was the suggested treatment. The three-stage Mayo Clinic or the two-stage Mickulicz procedures gradually replaced this. Fears of inadequate control of the source of sepsis prompted the implementation of the resection of the affected segment of colon with formation of a colostomy (HP) in the 1970’s. The future development of the treatment strategies was driven by the recognition of the high morbidity and mortality associated with HP and the low Hartmann’s reversal rates. This led to the wider use of resection with PRA during the 1990’s.

The technique of lavage and drainage regained its popularity during the 1960’s. It has relatively recently become possible to perform this procedure laparoscopically which takes advantage of the benefits of minimally invasive surgery with faster recovery and shorter hospital stay. Using this strategy allows resection surgery to be postponed or avoided altogether in many patients; moreover, an higher rates of PRA can be achieved avoiding the need for a stoma. The three recent RCTs of LPL reported inconsistent outcomes. These findings warrant further research and debate.
